# CTCF Genomic Binding Sites in *Drosophila* and the Organisation of the Bithorax Complex

**DOI:** 10.1371/journal.pgen.0030112

**Published:** 2007-07-06

**Authors:** Eimear E Holohan, Camilla Kwong, Boris Adryan, Marek Bartkuhn, Martin Herold, Rainer Renkawitz, Steven Russell, Robert White

**Affiliations:** 1 Department of Physiology, Development and Neuroscience, University of Cambridge, Cambridge, United Kingdom; 2 Institute for Genetics, Justus-Liebig-University Giessen, Giessen, Germany; 3 Department of Genetics, University of Cambridge, Cambridge, United Kingdom; North Carolina State University, United States of America

## Abstract

Insulator or enhancer-blocking elements are proposed to play an important role in the regulation of transcription by preventing inappropriate enhancer/promoter interaction. The zinc-finger protein CTCF is well studied in vertebrates as an enhancer blocking factor, but *Drosophila* CTCF has only been characterised recently. To date only one endogenous binding location for CTCF has been identified in the *Drosophila* genome, the *Fab-8* insulator in the *Abdominal-B* locus in the Bithorax complex (BX-C). We carried out chromatin immunopurification coupled with genomic microarray analysis to identify CTCF binding sites within representative regions of the *Drosophila* genome, including the 3-Mb *Adh* region, the BX-C, and the Antennapedia complex. Location of in vivo CTCF binding within these regions enabled us to construct a robust CTCF binding-site consensus sequence. CTCF binding sites identified in the BX-C map precisely to the known insulator elements *Mcp, Fab-6,* and *Fab-8.* Other CTCF binding sites correlate with boundaries of regulatory domains allowing us to locate three additional presumptive insulator elements; “Fab-2,” “Fab-3,” and “Fab-4.” With the exception of *Fab-7,* our data indicate that CTCF is directly associated with all known or predicted insulators in the BX-C, suggesting that the functioning of these insulators involves a common CTCF-dependent mechanism. Comparison of the locations of the CTCF sites with characterised Polycomb target sites and histone modification provides support for the domain model of BX-C regulation.

## Author Summary

There is still much to learn about the organisation of regulatory elements that control where, when, and how much individual genes in the genome are transcribed. Several types of regulatory element have been identified; some, such as enhancers, act over large genomic distances. This creates a problem: how do such long-range elements only regulate their appropriate target genes? Insulator elements have been proposed to act as barriers within the genome, confining the effects of long-range regulatory elements. Here we have mapped the locations of one insulator-binding protein, CTCF, in several regions of the *Drosophila* genome. In particular, we have focussed on the Hox gene cluster in the Bithorax complex; a region whose regulation has been extensively characterised. Previous investigations have identified independent regulatory domains that control the expression of Bithorax complex genes in different segments of the fly, however the molecular nature of the domain boundaries is unclear. Our major result is that we find CTCF binding sites precisely located at the boundaries of these regulatory domains, giving a common molecular basis for these boundaries. This provides a clear example of the link between the positioning of insulators and the organisation of gene regulation in the Drosophila genome.

## Introduction

Insulator elements are DNA sequences that regulate interactions between promoters and enhancers. By preventing inappropriate enhancer/promoter communication, insulators are believed to play a key role in the genomic organisation of transcriptional regulation. Their mode of action is still unclear but may involve the formation of chromatin loops that partition the genome into separate regulatory domains [1–5].

In vertebrates, almost all characterised insulator elements are associated with the binding of CTCF, a DNA-binding protein that contains multiple zinc fingers. Although CTCF was initially identified as both a transcriptional activator and repressor [6–8], it was subsequently recognised as being essential for the enhancer blocking activity of several vertebrate insulators [9]. CTCF also functions in imprinting [10,11] and has been implicated in human disease [12]. Recently, *Drosophila* CTCF has been identified [13], joining other known *Drosophila* enhancer blocking proteins such as Su(Hw) [14], Zw5, and BEAF32 [15,16].

In addition to insulation of entire genes or groups of genes, insulators may also flank individual enhancers allowing them to act independently, facilitating complex tissue and cell-specific patterns of gene expression [17]. This function is particularly relevant in the case of the Hox genes, whose complex expression patterns specify segmental identities along the body axis. In *Drosophila*, correct antero-posterior patterning in the thorax and abdomen is dependent on the precise expression of the Hox genes of the Bithorax complex (BX-C) in specific parasegments [18,19]. This is achieved by the subdivision of the regulatory regions of each of the three BX-C genes (*Ultrabithorax [Ubx], abdominal-A [abd-A],* and *Abdominal-B [Abd-B]*) into distinct enhancer domains [20]. There are at least nine distinct regulatory regions, each important for specifying homeotic gene expression in individual thoracic and abdominal parasegments (PS) from PS 5–13 [21–25]. The domain hypothesis of Mihaly et al. [26] proposes that each distinct regulatory region or domain contains a modular arrangement of functional elements required for Hox gene expression in a particular parasegment. These elements include initiator, enhancer, and memory elements/Polycomb-response elements (PREs). It is thought that boundary elements, located between adjacent domains, restrict the influence of each regulatory region. The evidence for this comes from mutations that disrupt boundary function and from enhancer trap transposon studies, which have generated a map of the BX-C compartmentalised into distinct parasegmental regulatory regions [27,28]. Three boundaries *Mcp, Fab-7,* and *Fab-8* have been defined by mutation [29–33]. Another, *Fab-6,* has been mapped genetically [26], and others are postulated to exist. Each of the three BX-C boundaries identified by mutational analysis display insulator function; i.e., they are capable of suppressing reporter gene expression when placed between an enhancer and a promoter in a transgenic insulator assay [4,29,34–36]. Recently, Moon et al. [13] showed that the *Fab-8* boundary element contains binding sites for CTCF and that mutation of these sites greatly reduces the ability of *Fab-8* to suppress reporter gene expression in an insulator assay, demonstrating that the insulating activity of *Fab-8* is dependent on CTCF.

Here we use chromatin immunopurification together with genomic microarray (ChIP-array) to investigate in vivo CTCF binding in several regions of the *Drosophila* genome, including the BX-C. From this analysis, we identify a CTCF binding-site consensus that allows the precise location of CTCF binding sites in these genomic regions. In the BX-C, in addition to the characterised CTCF sites in the *Fab-8* boundary element, we demonstrate the presence of CTCF binding sites in the *Mcp* and *Fab-6* boundaries. Furthermore, we identified CTCF binding sites between the regulatory regions *bxd/pbx* and *iab-2,* between *iab-2* and *iab-3,* and between *iab-3* and *iab-4,* providing both a localisation of the previously postulated boundary regions of “Fab-2,” “Fab-3,” and “Fab-4” and a demonstration that these too bind CTCF.

A number of CTCF binding sites have been identified in the vertebrate genome, but there is little agreement as to the similarity between these sites at the DNA level [7,13,37]. Binding data have been interpreted to suggest that different combinations of zinc fingers are used to bind to differing sites of approximately 50 bp [7,38]. In contrast, our analysis in *Drosophila* indicates that CTCF sites contain a conserved consensus binding sequence of approximately 20 bp in length. Examination of the vertebrate CTCF binding sites reveals that they too contain a consensus sequence and that this vertebrate CTCF consensus is similar to the *Drosophila* site identified here.

## Results

### Identification of In Vivo CTCF Binding Locations

In order to identify the in vivo binding sites of the *Drosophila* CTCF protein, we used our previously described ChIP-array procedure [39]. Sonicated chromatin, isolated from *Drosophila* embryos, was immunopurified using either anti-CTCF antiserum (specific immunopurification [IP]) or normal rabbit serum (control IP). The immunopurified DNA preparations were labelled with either Cy3 or Cy5 and hybridised to a 1-kb tiling-path genomic microarray covering the 3-Mb *Adh* region together with other selected genomic regions including the BX-C, the Antennapedia complex (ANT-C), and the *achaete-scute* region. As a positive control, the immunopurification reactions were assessed using specific PCR primers to amplify a 378-bp fragment from the *Fab-8* region, containing characterised CTCF binding sites [13]. This fragment showed clear enrichment when compared with amplification using primers for a 300-bp fragment (Clone 10) that does not contain a CTCF binding site (unpublished data).

**Figure 1:**
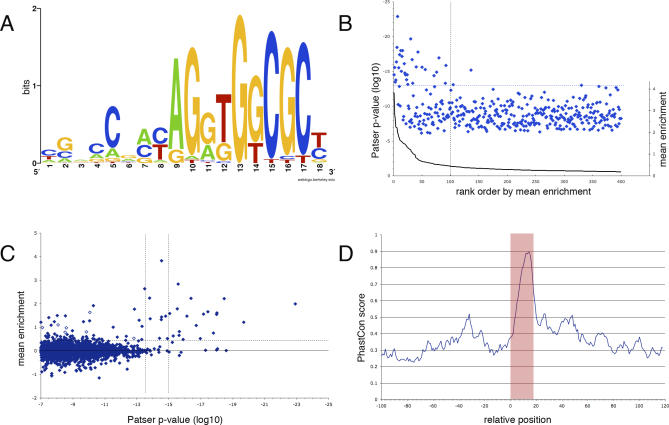
*Drosophila* CTCF In Vivo Binding-Site Consensus (A) Shown is the sequence logo of the CTCF consensus. (B and C) Correspondence between ChIP enrichment and closeness of match to the CTCF consensus is presented. (B) The top 600 enriched fragments are ordered by Mn (equivalent to log_2_ratio, black line), and the corresponding Patser *p*-values are plotted (blue diamonds). The blue horizontal dotted line indicates Patser *p* < 10^−13^, which approximately marks the boundary of the *p*-value distribution associated with nonenriched fragments. The black vertical dotted line indicates the position in the rank order of Mn = 0.45. (C) The lowest Patser *p*-value for each fragment is plotted against the Mn for that fragment (blue diamonds). Open diamonds indicate fragments with *p* > 10^−13^ that are neighbours to fragments with lower *p*-values. The horizontal dotted line indicates Mn = 0.45. Candidate enriched fragments were selected on Mn > 0.45 and CyberT *p* < 0.05. The vertical dotted lines indicate the positions of Patser *p* = 10^−13.5^ and 10^−15^. (D) PhastCons scores are shown across all 855 predicted genomic CTCF binding sites with a Patser *p* < 10^−15^. The binding sites are centred over position 0, and 100 bp left and right of the site are shown. The blue line indicates the median PhastCons score for a given position. There is a prominent peak corresponding to the CTCF motif. The flanking sequences show some minor fluctuations in conservation of unclear significance.

Replicated hybridisation to genomic DNA tiling arrays generated a dataset (Dataset S1) with mean enrichments (Mn) equivalent to log_2_ 3.8 (14-fold) observed. The *Fab-8* positive control is represented on the array as fragment UBX65, which gave an enrichment value of 1.56 (3-fold) and good reproducibility (*p* = 0.0045 across four biological replicates). Fragments showing Mn > 0.45 (1.4-fold) and *p* < 0.05 were selected as potential CTCF binding sites (Figure 1). A total of 33 fragments satisfied these criteria, including 18 from the 3-Mb *Adh* region, nine fragments from the BX-C, and four from the ANT-C (Dataset S2).

### Identification of a CTCF Binding Consensus

To identify potential CTCF binding sites within enriched fragments, the 33 candidate fragments were submitted to a motif discovery tool, Multiple Em for Motif Elicitation (MEME), to search for overrepresented sequence motifs [40]. The top motif found by MEME (*e* = 1.3 × 10^−20^) (Figure 1A) is identified in 23 out of the 33 fragments (70%). Of the remaining ten fragments, four are immediately adjacent to fragments that possess a match to this MEME motif. Of the 23 fragments that contain the top MEME motif, seven contain two sites resulting in a total occurrence of 30 sites. Acting as a positive control, both of the CTCF binding sites identified in the *Fab-8* region by DMS methylation interference [13] are identified by our MEME analysis. The 18-bp MEME motif is illustrated as a sequence logo in Figure 1A and shows rather weak 5′ sequence preference but a strong AGGTGGCGC consensus towards its 3′ end.

### Correlation between CTCF Binding Consensus and CTCF Binding

The 30 occurrences of the MEME motif were used to construct a position-specific weight matrix that was in turn used as the input for the Patser profile-matching tool to search for matches within the genomic sequences on the microarray.

The association between Patser matches and CTCF binding is demonstrated in Figure 1B and 1C. In Figure 1B the top 600 fragments are plotted in rank order by Mn along with the minimum *p*-value Patser site associated with each fragment. Clearly the high-ranking enriched fragments are generally associated with good matches to the CTCF weight matrix. Figure 1C shows that match to the CTCF weight matrix is a relatively good predictor of binding; of the fragments containing a better than *p* = 10^−13.5^ match, 57% have Mn > 0.45 (1.4-fold), and for *p* < 10^−15^, 70% have an enrichment >0.45. At a whole-genome level, we find 855 matches (*p* < 10^−15^) when Patser is used to search the Drosophila melanogaster genome sequence.

Another way to examine the functional relevance of these predicted sites is to look at their conservation across species. Figure 1D shows the conservation across 14 insect species aligned with D. melanogaster for the 855 matches within the *Drosophila* genome with Patser *p* < 10^−15^, plotting the median conservation across the CTCF binding motif together with 100 bp of flanking sequences. The plot shows a clear peak of conservation aligned with the 18-bp CTCF weight matrix matches. The conservation peak also appears to be approximately 20 bp wide and, as with the CTCF weight matrix, the conservation is greater towards the 3′ end of the motif. The conservation plot additionally suggests that conserved and hence potentially functionally relevant sequences may extend a few base pairs 3′ to the 18-bp CTCF binding motif.

Taken together, these data support the idea that the binding sites for CTCF in *Drosophila* can be described by a single weight matrix approximately 20 bp in length. This is clearly at odds with the notion, derived from studies of CTCF DNA binding in vertebrates, that CTCF binds to 50-bp target sites with a diverse spectrum of sequences [7,38].

### CTCF Sites in the Bithorax Complex

**Figure 2:**
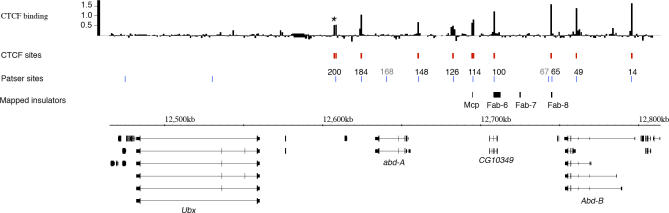
CTCF Binding Profile across the BX-C The top track shows the CTCF Mn per fragment across the region. Black asterisk marks fragment UBX200. CTCF sites are sites with Patser *p* < 10^−13^, which also show significant enrichment (mean > 0.45; *p* < 0.05, red bars). Patser sites are positions of Patser matches to CTCF consensus with *p* < 10^−13^ (blue bars). The numbers above the blue bars relate the Patser sites to the fragments used in the validation ChIPs and EMSA (Figure 3); the sites 67 and 168 (grey) are not associated with significant enrichment. The positions 148, 100, and 65 have closely spaced double sites. The positions of the mapped insulator elements are indicated above the sequence coordinate line and RefSeq genes below. Enriched fragments correlate well with Patser sites and with the positions of three of the mapped insulators; *Mcp, Fab-6,* and *Fab-8*.

The ChIP-array analysis identifies eight locations with CTCF binding within the BX-C. As shown in Figure 2, these eight locations show a striking correspondence with Patser CTCF site predictions as all eight locations overlap sites with a Patser *p* < 10^−13^. In addition, another UBX fragment (UBX200; Mn = 0.54 and *p* = 0.054) that falls just short of the significance threshold also contains a high-scoring Patser site (*p* = 10^−14.4^). ChIP enrichment of this fragment was validated by specific PCR, and this location was therefore included in the list of CTCF occupied sites, resulting in the identification of nine CTCF binding regions across the BX-C as depicted in Figure 2. Of these regions six contain a single *p* < 10^−13^ Patser match and three contain a pair of sites, separated by less than 200 bp (Table 1).

**Table 1:**
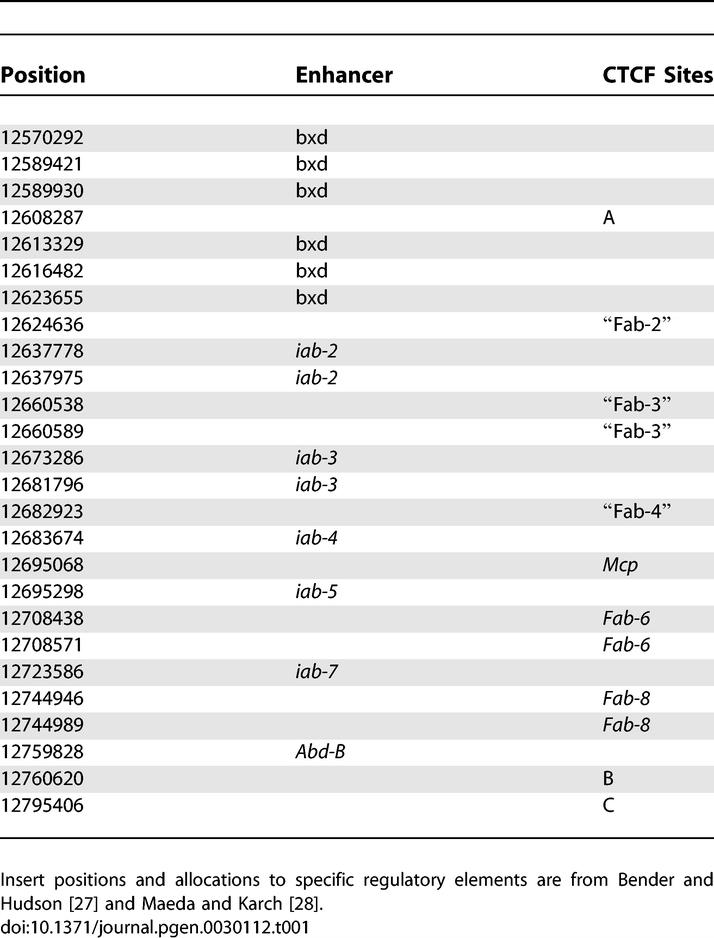
Coordinates of CTCF Sites and Enhancer Trap Inserts in the BX-C

**Figure 3:**
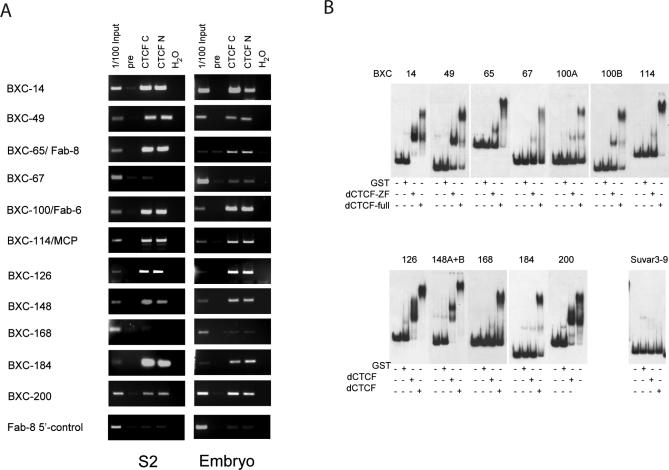
Validation of CTCF Sites by ChIP and In Vitro Binding (A) ChIP was performed with chromatin from *Drosophila* Schneider cell line 2 (S2) cells and from embryos. Fragments are numbered according to coordinates from the original *Drosophila* Genome Project sequencing of the BX-C (see Figure 2) [20]. CTCF-specific antibodies (C- or N-terminal specific) immunopurify the same set of CTCF binding sites as were enriched in the ChIP-array. Negative controls were pre-immune serum or a nonbinding sequence (*Fab-8* 5′-control). BXC-67 and 168 show very weak, if any, enrichment. (B) In vitro binding assays (EMSA) show direct binding of CTCF to predicted CTCF sites in the BX-C. Radioactively labelled probes were incubated with GST, GST-CTCF-ZF, or GST-CTCF full length. All predicted sites are bound. A negative control sequence from the *Su(var)3–9* gene does not bind to CTCF.

We performed ChIP experiments with crosslinked chromatin from both *Drosophila* S2 cells and embryos to validate this set of CTCF sites (Figure 3A). To control the specificity of the immunopurifications, we used two different CTCF antibodies, raised against the N- or C-terminal part of the protein. For negative controls we used pre-immune serum as well as a nonbinding sequence, encompassing a sequence upstream of *Fab-8* (*Fab-8* 5′-control). The results indicated association of CTCF with the same set of fragments that were enriched in the ChIP-array. A total of two fragments with strong Patser matches, but no enrichment in the ChIP-array (BXC-67 and BXC-168), also showed no clear enrichment in the fragment-specific ChIP assay.

Since the nine CTCF binding regions show both ChIP enrichment and high-scoring Patser matches, they are likely to be direct CTCF targets rather than products of indirect association through, for example, chromatin looping. To substantiate this we analysed DNA binding in vitro by electrophoretic mobility shift assay (EMSA) with radioactively labelled probes and bacterially expressed purified GST-CTCF fusion proteins (Figure 3B). We used either the 11-zinc-finger DNA-binding domain or the full-length protein fused to GST, which differentially retard the DNA fragments. The GST domain by itself does not bind, nor did a negative control sequence from *Su(var)3–9* bind to CTCF. These experiments show direct binding of CTCF to all of the sites predicted by the Patser analysis, even to BXC-67 and BXC-168, which were very weakly, if at all, enriched by ChIP. Some of the sites were bound by the full-length protein, but not by the isolated zinc-finger-region of CTCF. This may suggest that other amino acids in regions outside of the zinc-finger region participate in DNA binding of CTCF. The double sites at BXC-148 (A + B) cannot be resolved on two different fragments, rather single and double occupancy can be seen with the zinc-finger domain causing two different shifted bands. Overall, our data strongly indicate that the CTCF sites we have identified in the BX-C are direct CTCF binding sites.

As shown in Figure 2, we find a striking correspondence between in vivo CTCF binding and mapped boundary elements in the BX-C. In addition to the known binding of CTCF at the *Fab-8* insulator [13], we show that CTCF binding is also detected within the mapped domains of the *Mcp* and *Fab-6* boundary elements.

The remaining mapped boundary, *Fab-7,* shows neither significant CTCF binding in the ChIP-array analysis nor a Patser site *p* < 10^−13^. However, using the more sensitive PCR assay of ChIP enrichment, we do observe a relatively weak but significant association of CTCF with *Fab-7* (Figure S1).

**Figure 4:**
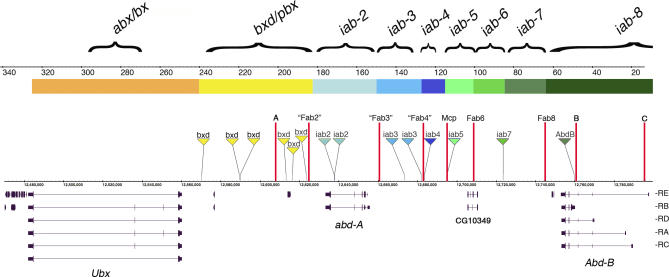
CTCF Binding Sites Demarcate Boundaries in the BX-C The top track shows the BX-C *cis*-regulatory regions above a coordinate line with numbering according to Martin et al. [20]. The coloured bar indicates the regulatory domains according to Maeda and Karch [28]. The orange/yellow regions *(abx/bx* and *bxd/pbx)* control *Ubx* expression. The blue shaded regions *(iab-2*, *3,* and *4)* control *abd-A* expression, and the green regions *(iab-5* to *9)* regulate *Abd-B*. Above the *Drosophila* genome Release 4 coordinate line, are shown the insertion points and associated regulation of a set of enhancer-trap insertions [27]. The locations of CTCF sites are indicated by red vertical bars. The positions of the CTCF sites correlate well with the boundaries of the *cis*-regulatory regions and separate sets of enhancer-traps with different regulation.

Given the strong connection of CTCF sites to mapped boundary elements, we investigated whether the other CTCF sites within the BX-C also identified boundaries. The positions of boundary elements can be estimated from the mapping of mutations that affect the individual parasegment-specific regulatory elements, and the extents of these *cis*-regulatory domains (taken from Maeda and Karch [28]) are indicated by the coloured bar in Figure 4. Again we find a clear correspondence; CTCF sites are located close to the boundaries between bxd/pbx and *iab-2* (the “Fab-2” boundary), between *iab-2* and *iab-3* (the “Fab-3” boundary), and between *iab-3* and *iab-4* (the “Fab-4” boundary). However, mapping of these regulatory domains can be imprecise, particularly if the mutations are chromosomal rearrangements with complex effects. A more robust map of the regulatory domains is provided by the locations of enhancer-trap insertions and the analysis of their patterns of expression [27,28]. The positions of 14 enhancer traps in the BX-C are shown in Figure 4 (coordinates in Table 1), together with their allocations to specific regulatory domains based on their expression patterns. Again we find that CTCF binding sites separate different regulatory domains. The location of the Fab-4 site is particularly compelling; enhancer traps detecting *iab-3* and *iab-4* regulation are separated by less than 2 kb, and this interval contains the “Fab-4” CTCF site.

Of the remaining three CTCF binding sites with the BX-C (sites A–C, Figure 4), two sites are within introns of *Abd-B* close to alternative *Abd-B* transcription start sites (Abd-B-RB and Abd-B-RE, respectively). The third site lies within the *bxd/pbx* regulatory region.

In summary, the CTCF sites identified here correlate with six out of seven known or postulated boundary elements, the only exception being *Fab-7.* As CTCF has been demonstrated to be required for insulator function at *Fab-8* [13], it is likely that all these CTCF-associated boundaries function through a common CTCF-dependent mechanism.

### Genomic Context of CTCF Sites in the Bithorax Complex

According to the domain model of BX-C regulation, the domains bounded by insulators would act as autonomous units that could either be active or silenced depending on the state of memory elements/PREs within each domain [26]. This is likely to require a precise arrangement of insulators and PREs to restrict PRE-dependent chromatin modification to specific domains. Several PREs have been mapped within the BX-C and, in particular, PREs have been located close to the boundary elements *Mcp*, *Fab-7,* and *Fab-8* [29,33,41]. We were interested in examining the relationships between CTCF sites, the binding sites for Polycomb complexes, and the domains of chromatin modification. For this analysis we compared our CTCF ChIP-array data with a genome-wide analysis of Polycomb targets in *Drosophila* that determined the genomic binding sites for Polycomb Repressive Complex 1 (PRC1) complex components (Pc and Psc), for the Polycomb Repressive Complex 2 (PRC2) complex component E(Z), and for the PRC2-dependent chromatin modification, trimethylation of histone H3 lysine 27 (H3K27me3), in S2 cells [42]. In this particular cell line the *Abd-B* gene is expressed; the four downstream *Abd-B* promoters are active, but the most upstream promoter (*Abd-B*-RE) is silenced. Schwartz et al. [42] found that the *Fab-7* and *Fab-8* PREs are not bound by Polycomb complexes, and the *Abd-B* transcription unit is largely within an “open” domain devoid of H3K27me3 histone modification.

**Figure 5:**
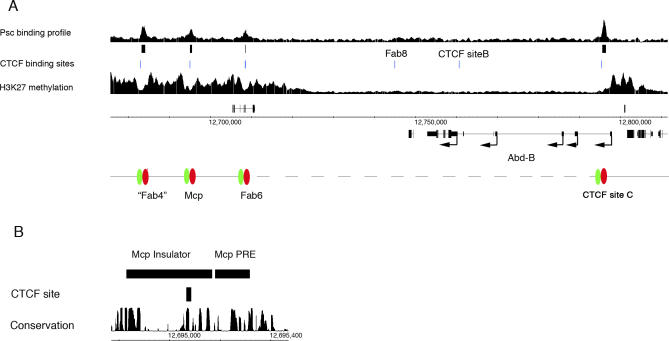
Genomic Context of CTCF Sites in the BX-C (A) Shown is a comparison between the locations of CTCF binding sites, Polycomb target sites, and histone H3 lysine 27 (H3K27) methylation from the data of Schwartz et al. [42]. For the Polycomb targets the Psc track is shown but the Pc and E(Z) binding profiles identify the same targets sites in this region. CTCF sites are closely related to Polycomb targets sites as illustrated by the schematic with CTCF sites in green and Polycomb sites in red. (B) Detailed view of the *Mcp* region shows the relationship between the CTCF site, the mapped domains of the insulator, the PRE, and the PhastCons conservation track. The CTCF site sits within the mapped insulator and lies over a clear discrete conservation peak.

In Figure 5, we display the relationship between these Polycomb data [42] and our in vivo CTCF binding data in the region from “Fab-4” to the 5′ end of the *Abd-B* transcription unit. Strikingly, the four Polycomb target sites in this region that are occupied by Polycomb complexes in S2 cells are all located in close proximity to CTCF binding sites. Furthermore, the Polycomb target peak always lies to one side of the CTCF site suggesting the relative arrangements as indicated in the schematic in Figure 5A. However, although the CTCF sites are precisely located, the Polycomb target sites are represented by peaks that span several hundred base pairs leaving some uncertainty as to the precise location of the Polycomb target sequences. Nevertheless, for *Mcp* this ordering agrees with the functional mapping where the PRE and the boundary have been mapped to adjacent but separate regions as illustrated in Figure 5B [4,41]. Overall, this arrangement suggests that each of these Polycomb target site sits within a domain flanked by CTCF boundaries.

The H3K27me3 profile also shows a relationship to the location of CTCF sites. The most prominent feature of the H3K27me3 profile in S2 cells is the domain between approximately 12,725,000 and 12,795,000, which lacks the repressive trimethylation of lysine 27 (K27me3) modification. The right-hand side of this domain has a sharp border that corresponds well with the CTCF site “C” at 12,795,406. The left-hand side of the domain does not have a clear border and does not fit with a CTCF site. It is tempting to speculate that the differences in the two borders of the H3K27me3 domain may be related to the relative arrangement of the CTCF and Polycomb sites. On the left-hand side, the Polycomb site is “outside” the CTCF site, and the H3K27me3 modification spreads rightwards from the Polycomb site. On the right-hand side, the Polycomb site is “inside” the CTCF site, and the H3K27me3 modification does not spread past the CTCF site. We also note that the positions of the CTCF site/PREs at “Fab-4,” *Mcp,* and *Fab-8* are associated with pronounced depressions in the K27me3 profile. This may be related to nucleosome depletion at PREs [43], but it is interesting that CTCF binding sites in the mouse ß-globin locus are also depleted for repressive chromatin marks [44].

We examined the conservation of the CTCF sites in the BX-C. The sites show high conservation with median PhastCons scores close to 1.0 across the approximately 20-bp motif. We illustrate this for an individual site *Mcp* (Figure 5B), where the single CTCF site corresponds to one of the clearly defined peaks of conservation that lie within the functionally mapped *Mcp* boundary element.

### CTCF Binding in Other Genomic Regions

**Figure 6:**
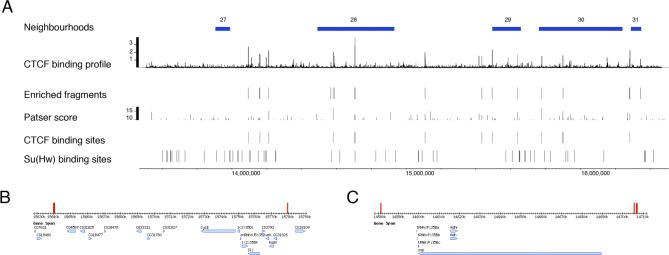
CTCF Sites across the 3-Mb Adh Region (A) CTCF binding profile shows the Mn (equivalent to log_2_ratio) for the array fragments. Enriched fragments row plots fragments with enrichment (mean > 0.45; *p* < 0.05). Patser score plots the scores for Patser hits with *p* < 10^−12^. CTCF binding sites depict Patser sites with *p* < 10^−12^, which are associated with significant enrichment (Mn > 0.45; *p* < 0.05). SuHw binding sites show the Patser *p* < 10^−15^ Su(Hw) binding sites from (B. Adryan, G. Woerfel, I. Birch-Machin, S. Gao, M. Quick, L. Meadows, S. Russell and R. White; unpublished data). There is no clear correlation between Su(Hw) sites and CTCF sites. Neighbourhoods row depicts the gene expression neighbourhoods of Spellman and Rubin [48]; the neighbourhood boundaries in some instances map close to CTCF sites, but the overall correspondence is not compelling. (B and C) Selected regions show the arrangement of CTCF sites (marked by red bars) around the *CycE* gene and the *osp* locus.

Other genomic regions screened for CTCF binding sites on the microarray include the 3-Mb *Adh* region and the smaller *Antennapedia* and *achaete-scute* regions*.* The *Adh* region [45] is a well-characterised region of Chromosome 2L, containing approximately 250 genes from *kuzbanian* to *cactus,* which serves as a representative region of the fly genome. The ChIP-array identified 18 fragments in the *Adh* region with Mn > 0.45 (1.4-fold) and *p* < 0.05 (Figure 6). Of these fragments, 11 contain high-scoring Patser CTCF weight-matrix matches (Patser *p*-value < 10^−12^), and two additional fragments, ADH-1602 and ADH-2233, are associated with flanking high-scoring Patser sites at distances of only 40 and 100 bp, respectively. In both cases these high-scoring Patser sites lie between two adjacent array fragments and so were not included as hits in the MEME analysis. PCR validation shows clear enrichment in both these cases (unpublished data). It was found that two fragments that lack strong Patser sites are neighbours of highly enriched fragments that do contain high-scoring Patser sites. ADH-2253 has an additional high scoring hit in a neighbouring fragment that did not show up as being enriched on the array. Overall, this results in a strong association between ChIP-array enrichment and the presence of a high-scoring Patser site, with a total of 15 out of 19 fragments possessing either a high-scoring Patser site or immediately neighbouring a fragment with a high-scoring site. The enriched fragments are associated with 17 CTCF sites in total (Figure 6).

Identification of CTCF binding sites within the *Adh* region presented an opportunity to investigate the relationship between CTCF binding sites and annotated genome features. Given CTCF's well-documented insulating function, it seemed likely that most identified sites would be in intergenic regions and this proved to be the case. Of the 17 sites in the ADH region, 15 (88%) are present in intergenic regions. No sites overlap exons, but two sites present in ADH-705 overlap the 3′ UTR region of the protein kinase gene *smell-impaired 35A* (*smi35A*). Most sites occur as single isolated sites (65% are separated by at least 500 bp), but there are three pairs of sites that are closer than 200 bp apart. Thus, in general, the CTCF sites in this region are not present in multisite clusters, but there are some closely spaced pairs of sites. Neighbouring sites, in general, flank several transcription units (e.g., the sites flanking *CyclinE [CycE]* shown in Figure 6B), but we also note that the long transcription unit of *outspread* (*osp),* which extends for about 90 kb, is rather neatly flanked by a pair of 5′ CTCF sites and a single 3′ site (Figure 6C).

We compared the location of CTCF sites with the sites we have identified for another *Drosophila* insulator-binding protein, Su(Hw) (B. Adryan, G. Woerfel, I. Birch-Machin, S. Gao, M. Quick, L. Meadows, S. Russell, and R. White; unpublished data). The Su(Hw) sites are illustrated in Figure 6, and we find no clear general relationship between Su(Hw) and CTCF sites. Only one fragment contained both Su(Hw) and CTCF sites (ADH-3002; 14,706,282–14,707,212). It is curious that this fragment was the top enriched fragment in both ChIP-array analyses. We also looked for an association between CTCF sites and gene neighbourhoods since insulators might provide a molecular basis for the occurrence of clusters of similarly expressed genes in the genome [46–48]. In Figure 6 we illustrate the gene neighbourhoods in the *Adh* region identified by Spellman and Rubin [48]. Although neighbourhood 29 is precisely flanked by CTCF sites, and there is a site separating neighbourhoods 30 and 31, overall we do not see a compelling association between CTCF sites and these gene expression neighbourhoods.

A total of three out of the four ChIP-array enriched fragments in the *Antennapedia* region displayed a match to the top motif discovered by MEME. The remaining fragment is a neighbouring fragment. All three directly enriched fragments contain at least one high-scoring Patser site (*p* < 10^−12^). In total, four sites are identified in the *Antennapedia* genomic region, and only one of the sites occurs in an intergenic region (ANT297). The remaining three sites are located within the first intron of *Antennapedia* itself. These sites consist of a pair of sites, 179 bp apart, and one “single” site. Only a single fragment was identified within the *achaete-scute* complex, this contains a high-scoring Patser site (*p* = 10^−14.5^) and is present in the intergenic region between *scute (sc)* and *lethal of scute (l(1)sc)*.

### Vertebrate CTCF Binding Site

**Figure 7:**
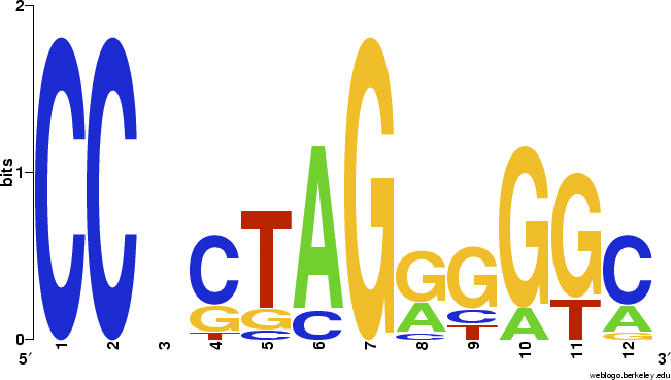
Vertebrate CTCF Consensus Shown is the sequence logo of a CTCF consensus sequence derived from sites collated in Moon et al. [13]. Both the vertebrate and the *Drosophila* motifs share the AGGNGGC consensus sequence, and the strong CC at positions1,2 in the vertebrate motif corresponds to the weaker CC preference at positions 4,5 in the *Drosophila* motif.

Although the existence of a region of similarity within different vertebrate CTCF binding sites has been noted [9,49], a consensus binding site has not been universally recognised, mainly because of experiments that suggest that CTCF binds to DNA by employing varying combinations of different zinc fingers [7,50,51]. Following identification of the *Drosophila* CTCF consensus binding site, we examined the possibility that the vertebrate and *Drosophila* binding sites are similar in sequence. We utilised the selection of sites compiled by Moon et al. [13] and submitted these sequences to the Motif Discovery tool, MEME. The highest scoring motif identified (*e* = 6.6 × 10^−11^) was found in all 12 sequences and is similar both to the conserved region identified previously in footprinting experiments and also to the *Drosophila* CTCF binding site reported here (Figure 7). Both the vertebrate and the *Drosophila* motifs share the AGGNGGC consensus sequence. Thus, our evidence does not support the idea that CTCF uses different combinations of zinc fingers to bind to different DNA sequences, and we suggest instead that CTCF binds to a similar specific sequence in both vertebrates and *Drosophila.*

## Discussion

The multiple zinc-finger DNA-binding protein CTCF is known to be required for the enhancer blocking action of vertebrate insulators, and a clear role for CTCF in the regulation of endogenous gene expression has been demonstrated at the imprinted Igf2 locus [9–11]. The mode of action of CTCF is, however, still unclear, although several studies have implicated CTCF in the formation of higher-order chromatin structure. CTCF molecules can interact to form clusters and thereby may mediate the formation of chromatin loop domains [44,52–54]. Partitioning of regulatory elements into independent chromatin loop domains is postulated to play a key role in the interactions between enhancers and promoters. Recently, a CTCF homolog was identified in *Drosophila,* and it was discovered that CTCF is required for the insulator function of the *Fab-8* element in the BX-C [13]. This observation opened up the prospect of utilising the wealth of genetic and molecular characterisation of BX-C transcriptional regulation for the analysis of CTCF function. Here we have used ChIP-array to investigate CTCF binding sites in regions of the *Drosophila* genome with a particular focus on the BX-C. We find that CTCF is not only associated with the *Fab-8* insulator, but also with other mapped boundary elements, *Fab-6* and *Mcp*. In addition, we show that CTCF sites are located at other postulated boundaries within the BX-C; “Fab-2,” “Fab-3,” and “Fab-4.” This provides a precise mapping of regulatory domain boundaries and a specific molecular foundation for the domain model of BX-C regulation.

We note that the *Fab-7* boundary may differ from the other characterised boundaries in the BX-C as we do not find a strong Patser match to the CTCF consensus in the functionally mapped *Fab-7* boundary element. Although *Fab-7* was not demonstrably enriched in the ChIP-array, we found significant CTCF association with *Fab-7* in the more sensitive PCR-base ChIP assay. Given the lack of a strong Patser match this may suggest an indirect association. We also do not see a CTCF site between the *abx/bx* and the *bxd/pbx* regulatory elements. However, these elements are separated by a long distance, and it is not clear whether they require insulation.

According to the domain model [26], the parasegment-specific regulatory domains that control the expression patterns of the *Ubx, abd-A,* and *Abd-B* genes of the BX-C are initially activated in appropriate parasegments by the early pattern-forming genes acting on initiator elements. Each regulatory domain is predicted to contain a particular initiator element, tuned to respond to a specific combination of gap and pair-rule gene products, thus activating the regulatory domain in the appropriate set of parasegments. This activation would be read by maintenance elements consisting of PREs that thereafter autonomously maintain each regulatory domain in either the OFF (silenced) or ON (active) state. Within a domain in the ON state, enhancers present in that domain would be able to engage with the relevant gene promoter and regulate expression of the gene. Boundary elements that flank each domain are proposed to restrict the effects of the initiator and maintenance elements to a single domain.

Although boundary elements are postulated to have the common property of insulating the regulatory domains, no sequence similarity between the mapped boundary elements has been reported until now. Here we show that a set of these boundary elements contain CTCF binding sites and bind CTCF in vivo. CTCF has been shown to be required for the insulator activity of *Fab-8,* and it seems likely that CTCF will also be a required component at the other boundary elements. In support of this suggestion, we find that the CTCF sites are well conserved within the sequenced insect genomes. The observation that CTCF sites flank a set of regulatory domains in the BX-C, together with the vertebrate studies that suggest that CTCF can mediate the formation of chromatin loops [44,52] supports the idea that interaction between CTCF sites may organise these domains into chromatin loops. However, how such a looping mechanism enables the autonomy of the individual regulatory domains and facilitates appropriate enhancer/promoter interactions is still unclear.

A key feature of the domain model is the relationship between the boundary and maintenance elements. For the domains to be capable of independently being set to the ON or OFF state, the range of influence of PREs needs to be restricted by the domain boundaries. Each domain would require at least one PRE. Our precise mapping of in vivo CTCF binding sites has enabled us to examine their relationship with Polycomb target sites. In strong support of the domain model, we find that the domains demarcated by CTCF sites contain Polycomb target sites. Indeed, we find an intimate relationship between CTCF and Polycomb binding sites as shown in Figure 5 for “Fab-4,” *Mcp, Fab-6,* and CTCF site “C.” This fits with previous functional mapping indicating that boundary elements and PREs are closely associated at *Fab-7, Fab-8,* and *Mcp*. This arrangement would impose a polarity on the spread of chromatin modification from the PRE, such that modification may start at the PRE abutting one boundary and spread across the domain in one direction towards the next boundary. At the boundaries, CTCF may play many possible roles. It could participate in boundary element function allowing the independence of chromatin domains by acting as a chromatin insulator blocking the spread of chromatin modification. However, at the chicken ß-globin locus, the chromatin boundary appears to be separable from the CTCF binding site [55]. Another possibility is suggested by that fact that CTCF has been demonstrated to block the progression of RNA polymerase [56]. This could potentially play an important role at boundaries in the BX-C to enable the independent function of PREs in neighbouring domains. There is considerable evidence that transcription through PREs may control their state, and many noncoding RNAs have been detected in the regulatory regions of the BX-C [56–62]. One role for CTCF could be to act as a barrier to such noncoding transcription, preventing transcripts arising in one regulatory domain from crossing into the neighbouring domain and affecting the PRE state. Such a role would be consistent with the observed location of CTCF sites in this region, as a CTCF site closely abuts one side of each PRE.

The individual regulatory domains must not only be able to act autonomously to set and maintain their activity state, but they must also be able to interact appropriately with the relevant gene promoters. Boundaries may play a role in this, and recently Cleard et al. [63] have demonstrated a long-range interaction between *Fab-7* and the *Abd-B*-RB promoter. This interaction was associated with lack of *Abd-B* expression, but similar interactions, bringing in appropriate enhancers, may also activate expression. The ability of CTCF to form clusters may facilitate such interactions, and it is intriguing that there are CTCF sites not only at the boundaries but also close to *Abd-B* promoters; the CTCF site “B” is 300 bp upstream of the *Adb-B*-RB promoter (Figure 4). Clustering of boundaries together with *Abd-B* promoter sequences may enable interaction between the promoter and enhancers in domains in the ON state. The clustering may also be more selective; in Figure 5 we see that in S2 cells, which specifically express *Abd-B*-RB, several boundaries are embedded in chromatin bearing the repressive H3K27me3 modification, whereas *Fab-8*, CTCF site “B,” and the *Abd-B*-RB promoter are in the unmodified, presumably “open,” chromatin domain. We could speculate that the expression of *Abd-B*-RB in these cells might be facilitated by interaction of the CTCF sites in the “open” domain, *Fab-8* and site “B,” enabling *Fab-8* to bring appropriate enhancers to the *Abd-B*-RB promoter.

We can compare this ChIP-array analysis of CTCF genomic sites with our ChIP-array analysis of binding sites for another *Drosophila* insulator-binding protein, Su(Hw) (B. Adryan, G. Woerfel, I. Birch-Machin, S. Gao, M. Quick, L. Meadows, S. Russell, and R. White; unpublished data). CTCF and Su(Hw) are both multi-zinc- finger DNA-binding proteins, and in both cases we have identified relatively long (~20 bp) consensus binding sites. In contrast to most DNA-binding proteins, we find that strength of match to the consensus binding sites is a good predictor of in vivo occupancy. We have also investigated whether our data indicate any collaboration between CTCF and Su(Hw). This seemed an attractive possibility since removing Su(Hw) function in vivo has little effect; *su(Hw)* null mutant flies are female-sterile but viable. Also, the insulating activity of *Fab-8* was significantly reduced when the CTCF sites were mutated but not completely abolished [13]. However we found no evidence for general colocalisation between CTCF and Su(Hw). A total of 60 Su(Hw) sites were identified in the *Adh* region, and only one of the fragments covering this region contained both CTCF and Su(Hw) sites. The single CTCF site identified in the *achaete-scute* complex was also some distance from the two Su(Hw) sites we found. Subsequent ChIP-array analysis in the BX-C led to the identification of only one Su(Hw) site within the entire BX-C region, in a location devoid of CTCF binding sites (B. Adryan, S. Russell, and R. White unpublished data). Indeed whilst the BX-C appears relatively enriched in CTCF sites compared to the *Adh* region, the converse is true for Su(Hw). For CTCF there are 4.7 sites/100 kb in the BX-C and 1.7 sites/100 kb in the Adh region (using Patser *p* < 10^−13^), whereas for Su(Hw) the BX-C is depleted in sites with only 0.29/100 kb in comparison to 2.7/100 kb in the Adh region (using Patser *p* < 10^−15^). Clearly, although CTCF and Su(Hw) both possess insulating ability, their sites of action do not correlate and there is no evidence from our analysis, covering approximately 3% of the *Drosophila* genome, for cooperative activity.

By comparing the sequences of ChIP-enriched fragments we identified a strong *Drosophila* consensus CTCF binding site. Analysis of vertebrate CTCF target sequences leads us to propose that vertebrate CTCF also binds to a similar consensus sequence. Our findings do not support the current view that CTCF binds to divergent DNA sequences by engaging different subsets of the zinc fingers [38,49,64]. Indeed, the binding site revealed here has been previously noted. Bell et al. [9] identified a CTCF binding site in the chicken β-globin insulator, and sequence comparisons between this site and other known CTCF sites [6–8] identified a conserved 3′ region, the mutation of which completely abolished CTCF binding and enhancer blocking. Filippova et al. [49] extended this comparison to include the Dm1 sites, mouse *H19* DMD4 and DMD7 and human *MYC A,* and again identified a conserved region within the larger approximately 50-bp DNase footprint for each site. It is this conserved region that corresponds to the vertebrate CTCF site found here. Very recently, an analysis of CTCF binding in the human genome has generated a vertebrate CTCF consensus site [65], and a CTCF consensus has also been derived from analysis of conserved regions in the human genome [66]. Both these sites are very similar to the consensus we identify here; in particular they share the strong features of the CC at positions 1 and 2, the AG at positions 6 and 7, and the GGC at positions 10, 11, and 12. Overall, these findings indicate that CTCF in both *Drosophila* and vertebrates binds to a single core consensus sequence.

In summary, ChIP-array analysis has enabled us to construct a CTCF binding site consensus. Mapping of genomic binding sites leads us to propose that all known or predicted insulators in the BX-C (with the possible exception of *Fab-7*) function in a CTCF dependent manner.

## Materials and Methods

### Fly strains and antibodies.

The wild-type strain used was OregonR. The primary antibody used was rabbit anti-CTCF-C [13].

### Chromatin isolation and immunopurification for microarray analysis.

Chromatin from embryos aged between 0 to 20 h after egg laying was purified as described previously [39]. The 300-μl immunopurification reaction contained 1.0 μl of rabbit anti*-*CTCF antibody for the specific IP or 1 μl of normal rabbit antiserum for the control IP. ChIP enrichment was assayed using PCR with specific primers as described previously [39]. The primers used were to *Fab-8* (UBX65), catcttccgttcatccgtttc and tgttggtgagcaagcgaaga, and Clone 10, attgggattctgcgattctg and tactgttcctggtgctggtg [13]. Validation ChIP assays for the CTCF sites in the BX-C were performed according to Moon et al. [13]. The validation ChIP primers are listed in Table S1.

### Microarray analysis.

The arrays used consist of 4,213 PCR products most of which are approximately 1 kb in length. The regions covered by the PCR products include the 3-Mb *Adh* region from *kuzbanian* to *cactus,* the *BX-C* and *ANT-C* regions, and 130 kb of the *achaete-scute* complex.

Amplification and labelling of DNA from enriched chromatin and hybridisations to genomic DNA tiling arrays were carried out as described previously [39]. We used four biological replicates (i.e., independent chromatin preparations), and each of these was hybridised as dye-swap technical replicates giving 16 array hybridisations in total. Microarray scanning, spot-finding, and normalisation were performed as described in Birch-Machin et al. [39] and on the FlyChip Web site (http://www.flychip.org.uk). The normalisation used VSN [67], which is based on an arsinh transformation and generates an enrichment measure that is generally equivalent to the log_2_ Cy3/Cy5 ratio. Statistical significance was assessed using the CyberT framework (http://visitor.ics.uci.edu/genex/cybert) [68].

### Binding site analysis.

The MEME version 3.0 Web site [40] was used to identify a consensus sequence. Parameters were set to discover up to six motifs between ten to 30 nucleotides in length. The consensus sequence for the CTCF binding motif was depicted using the MEME site stack in WebLogo (http://weblogo.berkeley.edu). The site stack for the CTCF binding motif was used to create a position-specific weight matrix (Table S2) for the Patser Web interface (http://rsat.ulb.ac.be/rsat/patser_form.cgi) [69]. This position-specific weight matrix was used to search DNA sequences present on the array and the *Drosophila* genome for matches to the consensus sequence using Release 4.0 coordinates. Patser generates a score for each position and provides a *p*-value; this is the probability of observing a particular score or higher at a particular sequence position. The Affymetrix Integrated Genome Browser (http://www.affymetrix.com/support/developer/tools/affytools.affx) was used to visualise the in vivo CTCF binding profile across the genome. Analysis of the evolutionary conservation of CTCF motifs used the PhastCons multiple alignment data available from the University of California Santa Cruz (Santa Cruz, California, United States) Genome Browser Web site using phastCons15way on D. melanogaster genome Release 4 (http://genome.ucsc.edu).

### EMSA.

Radiolabelled DNA probes (150–250 bp) were generated by PCR with ^32^P-labelled oligonucleotide primers and prepared by subsequent gel purification. The probes were incubated with 0.2 μg of purified GST, GST-CTCF, or GST-CTCF ZF. Recombinant proteins were prepared as described previously [70]. The binding reaction was performed in PBS ([pH 7.4], supplemented with 5 mM MgCl_2_, 1 mM ZnCl_2_, 1 mM DTT, 0.1% NP-40, and 10% Glycerol) for 15 min at room temperature in the presence of 200 ng/μl pdIdC. Protein-DNA complexes were analysed on nondenaturing poylacrylamide gels (3.5% acrylamide [w/v]) in TAE-buffer. Electrophoresis was performed at 4 °C with a field strength of 12 V/cm for 3 h.

## Supporting Information

Dataset S1 CTCF ChIP-Array Data Table shows the Array Spot ID, chromosome coordinates, Fragment ID, the values for the four biological replicate ratios, number of observations, Mn, standard deviation, *t*-value, and *p*-value derived by CyberT from the ChIP-array data.
(483 KB TDS)

Dataset S2 The 33 Candidate Enriched Fragments Table shows Fragment ID, start coordinate, stop coordinate, CyberT Mn, *t*-value, and *p*-value for the selected fragments with Mn > 0.45 and *p* < 0.05.
(13 KB PDF)

Figure S1 ChIP Analysis of CTCF Binding at *Fab-7*
ChIP was performed with chromatin from *Drosophila* S2 cells as in Figure 3 using CTCF-specific antibodies (C- or N-terminal specific). *Fab-8* is the positive control, and the negative controls were pre-immune serum or a nonbinding sequence (*Fab-8* 5′-control). *Fab-7* shows significant enrichment, although less enrichment than *Fab-8*.
(54 KB PDF)

Table S1 Primers for Validation ChIP Assays (29 KB DOC)

Table S2 CTCF Position-Specific Weight Matrix (12 KB PDF)

### Accession Numbers

The Gene Expression Omnibus (GEO) (http://www.ncbi.nlm.nih.gov/geo) accession number for the genomic tiling array is GEO Platform GPL5028 XC003 and for the ChIP data is series GSE7351.

The Entrez Gene (http://www.ncbi.nlm.nih.gov) accession numbers of the genes discussed in this paper are: *CTCF* human, 10664 and *Ctcf* mouse, 13018.

The Flybase (http://flybase.bio.indiana.edu) accession numbers of the genes and gene products discussed in this paper are: *abdominal-A (abd-A),* FBgn0000014; *Abdominal-B (Abd-B),* FBgn0000015; *achaete (ac),* FBgn0000022; *Alcohol dehydrogenase (Adh),* FBgn0000055; *Antennapedia (Antp),* FBgn0000095; BEAF32, FBgn0015602; *cactus (cact),* FBgn0000250; *CTCF*, FBgn0035769; *Cyclin E (CycE),* FBgn0010382; *Enhancer of zeste (E(z)),* FBgn0000629; *kuzbanian (kuz),* FBgn0015954; *lethal of scute (l(1)sc),* FBgn0002561; *outspread (osp),* FBgn0003016; *Polycomb (Pc),* FBgn0003042; *Posterior sex combs (Psc),* FBgn0005624; *scute (sc),* FBgn0004170; *smell-impaired 35A (smi35A),* FBgn0016930; *suppressor of Hairy wing (su(Hw)),* FBgn0003567; *Su(var)3–9,* FBgn0003600; *Ultrabithorax (Ubx),* FBgn0003944; and Zw5, FBgn0000520.

## Abbreviations

BX-C: Bithorax complex
ChIP: chromatin immunopurification
EMSA: electrophoretic mobility shift assay
H3K27me3: trimethylation of histone H3 lysine 27
IP: immunopurification
MEME: Multiple Em for Motif Elicitation
Mn: mean enrichment
PRE: polycomb-response element
