# Supplementary material for: CTCF Genomic Binding Sites in Drosophila and the Organisation of the Bithorax Complex
Source: PLoS Genet. 2007 Jul 6;3(7):e112. doi: 10.1371/journal.pgen.0030112 (PMC1904468; doi:10.1371/journal.pgen.0030112)
Supplement: Supplementary file 1 [file pgen.0030112.sd002.pdf]

Fragment ID	chromosome	nt_start	nt_stop	Mn	t	p
ADH-1075	chr2L	14581295	14582196	1.99312	6.36	0.0078509
ADH-1082	chr2L	14588278	14589212	1.49613	4.97	0.0156775
ADH-1602	chr2L	15109357	15110246	1.2463	3.19	0.0498795
ADH-1926	chr2L	15434332	15435254	1.47471	3.2	0.0493698
ADH-1986	chr2L	15494297	15495277	2.24399	7.54	0.00484489
ADH-2131	chr2L	15639318	15640245	1.65744	3.86	0.0308016
ADH-2233	chr2L	15779140	15780087	1.40726	4.83	0.0169044
ADH-2353	chr2L	15900287	15901211	0.536932	5.09	0.0146659
ADH-2734	chr2L	16282721	16283590	1.10872	7.28	0.0053501
ADH-2735	chr2L	16283737	16284648	2.22521	13.3	0.00091693
ADH-2797	chr2L	16345697	16346652	1.09445	6.07	0.00897571
ADH-2988(AD	chr2L	14568287	14569224	0.691022	3.62	0.0361577
ADH-3001(AD	chr2L	14705268	14706211	2.83217	15.2	0.0006187
ADH-3002(AD	chr2L	14706282	14707212	3.82697	22.5	0.00019135
ADH-588	chr2L	14094282	14095204	2.63725	23.2	0.00017584
ADH-654	chr2L	14160253	14161204	1.77116	3.36	0.0436216
ADH-655	chr2L	14161307	14162232	1.63658	3.44	0.041357
ADH-705	chr2L	14211282	14212217	2.22337	5.32	0.012962
ANT296	chr3R	2585618	2586544	0.987768	5.81	0.0101641
ANT297	chr3R	2584663	2585607	1.58982	7.44	0.00502906
ANT84	chr3R	2798559	2799535	1.77878	11	0.00160632
ANT87	chr3R	2795586	2796536	1.30526	3.68	0.0346727
as-c.47	chrX	263822	264870	1.43281	3.77	0.0326912
p9	chr2R	4662349	4663340	0.640185	12	0.00124103
UBX100	chr3R	12708202	12709142	1.21269	7.21	0.00549684
UBX113	chr3R	12695177	12696103	0.794967	7.49	0.00492347
UBX114	chr3R	12694232	12695108	0.637737	4.98	0.0155894
UBX126	chr3R	12682224	12683124	0.484172	4.69	0.0183625
UBX14	chr3R	12795207	12796155	1.60864	5.09	0.0146823
UBX148	chr3R	12660219	12661106	0.659504	14.4	0.00072548
UBX184	chr3R	12624208	12625144	1.04212	6.92	0.00619822
UBX49	chr3R	12760178	12761088	1.36248	5.89	0.00978868
UBX65	chr3R	12744228	12745136	1.55948	7.72	0.00452192
