# Supplementary material for: CTCF Genomic Binding Sites in Drosophila and the Organisation of the Bithorax Complex
Source: PLoS Genet. 2007 Jul 6;3(7):e112. doi: 10.1371/journal.pgen.0030112 (PMC1904468; doi:10.1371/journal.pgen.0030112)
Supplement: Supplementary file 2 [file pgen.0030112.sg001.pdf]

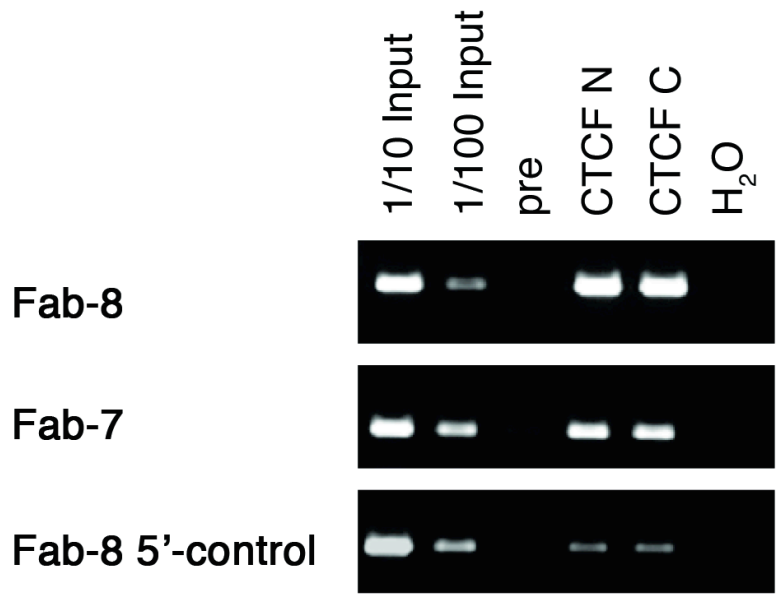
1/10 Input
1/100 Input
pre
CTCF N
CTCF C
H2O
Fab-8
Fab-7
Fab-8 5’-control
