# Supplementary material for: CTCF Genomic Binding Sites in Drosophila and the Organisation of the Bithorax Complex
Source: PLoS Genet. 2007 Jul 6;3(7):e112. doi: 10.1371/journal.pgen.0030112 (PMC1904468; doi:10.1371/journal.pgen.0030112)
Supplement: Supplementary file 3 [file pgen.0030112.st002.pdf]

A	6	5	11	7	5	9	14	2	26	1	9	0	0	0	0	0	0	1
C	14	8	4	16	22	4	11	14	0	0	1	1	0	0	29	1	28	11
G	2	16	10	5	2	13	5	1	4	28	19	8	30	23	0	28	0	3
T	8	1	5	2	1	4	0	13	0	1	1	21	0	7	1	1	2	15
