# Supplementary material for: CTCF Genomic Binding Sites in Drosophila and the Organisation of the Bithorax Complex
Source: PLoS Genet. 2007 Jul 6;3(7):e112. doi: 10.1371/journal.pgen.0030112 (PMC1904468; doi:10.1371/journal.pgen.0030112)
Supplement: Supplementary file 4 [file pgen.0030112.st001.doc]

**ChIP primer:**

BXC-14 f	CGA GTG CAC CCT CCT AGT CT

BXC-14 b	GAT TTA GAC TCC GCC CCT TT

BXC-49 f	ATC GAT AAA AAG CGC CAA CA

BXC-49 b	GCT CTT ACT GCC CGA TTC TG

Fab-8 f	GCA AGC GAA GAG TTC CAT TC

Fab-8 b	GGC ACA ATC AAG TTA ATG TTG G

Fab8 5' control f	CGA CGC TTT CAA TTC GTT TGT TTT

Fab8 5' control b	TTT GGA TAT CAC GGT GCT TTG GAT

BXC-67 f	GGA GCG CAT GAG ACA CTG TA

BXC-67 b	CTC GAT GGG GAT GTG TTT TC

BXC-100A f	TAT TCC ACC ACT CGG TCG TC

BXC-100A b	GCA GTT GCA GCT GAA GGA TA

BXC-100B f	TGG GAG ATA CAA AGA TAC CCA GA

BXC-100B b	CTA AAC CCG ATT TGC TTT GC

BXC-114 f	GGC TTT TCT GGG GAA GAA AT

BXC-114 b	GCG GCC ATG TAT TTT GTG TA

BXC-148A+B f	CCG TCC AAA CAT TTT TAC ACT TG

BXC-148A+B b	TGG CCA TCC AAT TAT TAC CG

BXC-168B f	TCG AGA ATG AGC GCA ATT TA

BXC-168B b	GCA AAT GCG TGG AAT TCT TT

BXC-184 f	TGA AAT CAA CTG GGA TTG TTC A

BXC-184 b	GAT CGG TGG CAT AAC CAT TC

BXC-200 f	CAT GGC AGA TAG GCA GGT TT

BXC-200 b	TCT GAC CCT GGA TTT TGC TC

Fab-7 f	CCA CTC GGT GTT CGC TCT ATT CAA

Fab-7 b	CAG GGG CAA GGC CGA ATG AA

Suvar39 f	AGC CGC TAC TAT TGC TTG GA

Suvar39 b	GCA GCG ACA GCA GTA TGA AA
